# Peptidic microarchitecture-trapped tumor vaccine combined with immune checkpoint inhibitor or PI3Kγ inhibitor can enhance immunogenicity and eradicate tumors

**DOI:** 10.1136/jitc-2021-003564

**Published:** 2022-02-25

**Authors:** Yang Du, Ye Liu, Di Wang, Hua Bai, Zhijie Wang, Xiran He, Pei Zhang, Jie Tian, Jie Wang

**Affiliations:** 1 CAS Key Laboratory of Molecular Imaging, Beijing Key Laboratory of Molecular Imaging, the State Key Laboratory of Management and Control for Complex Systems, Institute of Automation, Chinese Academy of Sciences, Beijing, China; 2 The University of Chinese Academy of Sciences, Beijing, China; 3 Institute of Medical Biology, Chinese Academy of Medical Sciences, Peking Union Medical College, Kunming, China; 4 Department of Medical Oncology, National Cancer Center/National Clinical Research Center for Cancer/Cancer Hospital, Chinese Academy of Medical Sciences & Peking Union Medical College, Beijing, China; 5 Beijing Advanced Innovation Center for Big Data-Based Precision Medicine, School of Medicine, Beihang University, Beijing, China; 6 School of Life Science and Technology, Xidian University, Xi'an, Shaanxi, China

**Keywords:** immunotherapy, immunogenicity, vaccine, tumor microenvironment, lymphocytes, tumor infiltrating, macrophages

## Abstract

**Background:**

With the rapid development of immune checkpoint inhibitors and neoantigen (NeoV)-based personalized tumor vaccines, tumor immunotherapy has shown promising therapeutic results. However, the limited efficacy of available tumor vaccines impedes the development of personalized tumor immunotherapy. In this study, we developed a novel tumor vaccine system and proposed combined therapeutic strategies for improving treatment effects.

**Methods:**

We developed a novel tumor vaccine system comprising a newly synthesized peptidic microarchitecture (PMA) with high assembly efficacy. The PMA-trapped neoantigen vaccine was developed to codeliver tumor neoantigen and the Toll-like receptor 9 agonist CpG (NeoV), abbreviated as PMA-NeoV. A microfluidic chip was used to produce PMA particles in a uniform and precise manner. Vaccine effectiveness was investigated both in vitro and in vivo. The combined immunotherapeutic effect of PMA-NeoV with anti-programmed cell death ligand 1 antibody (aPD-L1) or with the phosphatidylinositol 3‑kinase γ (PI3Kγ) inhibitor IPI-549 was further tested in MC38 mouse tumor model.

**Results:**

PMA-NeoV not only promoted codelivery of the tumor vaccine but also potentiated vaccine immunogenicity. Moreover, compared with free NeoV, PMA-NeoV significantly increased the number of tumor-infiltrating lymphocytes, promoted the neoantigen-specific systemic immune response, and suppressed murine colon MC38 tumor growth. Furthermore, PMA-NeoV increased the expression of programmed cell death receptor-1 on T lymphocytes, and in combination with aPD-L1 eradicated seven of eight MC38 tumors by rescuing exhausted T lymphocytes. Moreover, we combined the PMA-NeoV with the IPI-549, a molecular switch that controls immune suppression, and found that this combination significantly suppressed tumor growth and eradicated five of eight inoculated tumors, by switching suppressive macrophages to their active state and activating T cells to prime a robust tumor immune microenvironment.

**Conclusions:**

We developed a tumor vaccine delivery system and presented a promising personalized tumor vaccine-based therapeutic regimen in which a tumor vaccine delivery system is combined with an aPD-L1 or PI3Kγ inhibitor to improve tumor immunotherapy outcomes.

## Background

With rapid development of cancer immunotherapy, the patient’s own immune system is considered to play an important role in eliminating tumors. The two major objectives of immunotherapy are restoring the function of immunosuppressed immune cells using strategies such as immune checkpoint blockade (ICB) to recover the tumor-killing capability of immune cells^1^ and triggering systemic antitumor immunity by using treatments, such as chimeric antigen receptor T-cell immunotherapy and therapeutic tumor vaccines to amplify the tumor-specific immune response.^2 3^ Cancer therapeutic vaccines can treat cancer by activating patient’s immune system to recognize tumor-associated antigens to kill tumor cells.^4 5^ There are two main types of tumor antigens: whole tumor cell lysates (TCLs), which are weakly immunogenic because of their relatively small portion of tumor-specific antigens,^6–8^ and tumor-specific antigens, which are mutated proteins expressed in tumor cells and known as tumor neoantigens. Novel neoantigen vaccines have been developed for personalized cancer immunotherapy and evaluated in both preclinical studies and clinical trials.^4 9–14^ However, the response efficacies of neoantigen vaccines are seriously limited by insufficient delivery of the neoantigen peptide vaccine, preventing their clinical translation.^15^

To improve the delivery efficacy, attempts have been made to develop efficient tumor vaccine delivery strategies, mainly the following two strategies: (1) design nanomaterials as carriers to deliver neoantigen vaccines to overcome the fast degradation and controlled release of neoantigen vaccines, such as Montanide,^7 16^ or to manufacture nanoparticles of a suitable size to increase the trap efficiency of immune cells, such as nanodisks composed of synthetic high-density lipoprotein,^6^ multivalent bispecific nanobioconjugate engager, and synthetic polymeric nanoparticles^8 9^ and (2) use biological molecules as carriers to deliver neoantigen vaccines, such as albumin-based formulation DNA-RNA nanocapsules^11 14 17^ and DNA nanodevices.^17–19^ Although more efficient tumor nanovaccines are being developed, their maneuverability, safety, production quantity, and adverse effects greatly limit their applications.^20 21^ Peptidic carriers have received increased attention because of their advantages such as easy large-scale manufacture, controllable formulation preparation, and satisfactory biosafety.^22 23^ We recently reported that an oligopeptide can be assembled into a carrier to fundamentally enhance vaccine-induced immune responses in mice.^24 25^ The traditional synthesis process for fabricating microscale/nanoscale vaccine carriers includes emulsion, sonication, and purification of generated particles, requiring complex and multistep operations.^26^ Furthermore, the synthesized particles often have poor shape uniformity and size distribution, which may lead to poor replicability in vaccine delivery and release.^27^ However, this problem can be overcome by using a microfluidic chip to rapidly mix precursors inside microfluidic channels and precisely control the fluids, producing particles in a more rapid, uniform, and precise manner.^28 29^

To further increase the tumor vaccine response rate, a combination of ICBs has been used as a synergistic tumor therapy. A biadjuvant nanovaccine in combination with ICB was found to improve the immunogenicity of neoantigen vaccines and immunotherapeutic efficacy.^30^ Another clinical trial showed that neoantigen vaccine in combination with ICB dramatically enhanced therapeutic efficacy, providing promising therapeutic strategies for cancer management.^15^ However, the immunosuppressive tumor microenvironment consists of not only tumor cells but also non-tumoral stromal cells, including the highly notable tumor-associated macrophages.^31^ Recently, the mechanism underlying macrophage polarization has been widely examined, particularly the role of phosphatidylinositol 3-kinase γ (PI3Kγ), which functions as a molecular switch in the transformation of immunostimulatory M1 macrophages to immunosuppressive M2 macrophages.^32 33^ IPI-549, a small-molecule PI3Kγ inhibitor, selectively and potently inhibited PI3Kγ in animal studies with a favorable pharmacokinetic profile^32 34^ and good oral bioavailability and biosafety. Interestingly, IPI-549 caused no severe side effects in a phase I clinical trial (NCT02637531). Thus, combining IPI-549 with vaccine therapy may enhance therapeutic efficacy.

In this study, we developed peptidic microarchitecture (PMA) as a tumor vaccine carrier by constructing a new oligopeptide with significantly higher assembly efficacy and optimized its chemical structure. The microfluidic chip was used to rapidly produce more uniform and precise PMA particles. PMA can improve the codelivery of tumor vaccines and the Toll-like receptor (TLR) 9 agonist CpG to potentiate the immunogenicity of tumor antigens from both whole TCLs and neoantigen peptides to achieve potent and durable cancer immunotherapy. The combined immunotherapeutic effects of PMA-trapped neoantigens and CpG adjuvant (PMA-NeoV) vaccine with anti-programmed cell death ligand 1 (aPD-L1) or with the IPI-549 were further tested in MC38 tumor models. In general, we propose a tumor vaccine therapeutic system and strategy for cancer immunotherapy.

## Methods

### Materials and reagents

RPMI-1640 culture medium (Gibco, Grand Island, New York, USA), Dulbecco’s modified Eagle’s medium (Gibco), fetal bovine serum (FBS), penicillin-streptomycin (Gibco), recombinant granulocyte macrophage colony stimulating factor (GM-CSF, BioLegend, San Diego, California, USA), recombinant interleukin 4 (IL-4, BioLegend), lipopolysaccharide (LPS; eBioscience, San Diego, California, USA), CpG adjuvant (tlrl-1826; InvivoGen, San Diego, California, USA), collagenase type IV (Sigma-Aldrich, St. Louis, Missouri, USA), deoxyribonuclease I (Sigma-Aldrich), aPD-L1 (BioXcell, Beverly, Massachusetts, USA), IPI-549 (Selleckchem, Houston, Texas, USA), and an EasySep Mouse CD8^+^ T cell Isolation kit (STEMCELL Technology, Vancouver, Canada) were used.

### Synthesis and characterization of PMA-NeoV

The peptide (Nap-F-F-F-Y-p) was synthesized by GL Biochem (Shanghai, China). The peptide (10 mM) was dissolved in purified water at pH 8.5–9.5, after which 60 U/mL alkaline phosphatase (ALP) was added to formulate PMAs. After 30 min, we determined the structure of PMAs by analyzing their circular dichroism (CD) spectra (J-1500; JASCO, Oklahoma City, Oklahoma, USA). We used rheological measurements (Malvern Instruments, Malvern, UK) to confirm the formation of a viscoelastic stable peptidic assembly by detecting the storage modulus G′ and loss modulus G′′ of the peptidic assembly.

A microfluidic chip was used to manufacture PMA-NeoV. The device comprised three inlets, a straight microchannel, and an outlet. The fabrication process of PMA-NeoV is as following: the mixture of 10 mM oligopeptide (Nap-F-F-F-Y-p), 5 µg/mL CpG and 0.5 mg/mL neoantigen were dissolved in purified water at pH 8.5–9.5. ALP of 60 U/mL was used to catalyze the formation of as-prepared peptidic assembly-trapped NeoV. The syringe pump (Harvard Apparatus, Massachusetts, USA) was used to drive the injection of the as-prepared peptidic assembly into the microfluidic chip through inlet 1, and dichloromethane was simultaneously injected into inlets 2 and 3. The injection speed was 10 mL/hour. We collected the PMA-NeoV through the outlet. Dichloromethane can rapidly volatilize and leave the PMA-NeoV.

### Cell lines and cell culture

The murine colon cancer cell line MC38 and breast cancer cell line 4T1 were purchased from the National Infrastructure of Cell Line Resource (Beijing, China). The cells were cultured and maintained in RPMI-1640 supplemented with 10% FBS and 1% penicillin-streptomycin under 37°C in 5% CO_2_.

For bone marrow-derived dendritic cell (BMDC) culture, immature bone marrow cells were extracted from the tibias and femurs of 5-week-old C57BL/6J mice. First, red cells were removed by incubation with red cell lysis buffer. Second, bone marrow cells were washed and cultured in RPMI-1640 medium containing 10% FBS, 20 µg/mL GM-CSF, and 10 ng/mL IL-4 at a concentration of 1×10^6^ cells/mL. Third, half of the culture medium was replaced with fresh culture medium with the same concentration of GM-CSF and IL-4 every 2 days. On day 5, immature BMDCs were harvested for subsequent experiments.

For peripheral blood mononuclear cell (PBMC) culture, blood was obtained from the normal C57BL/6J mice using eyeball extirpating and stored in an anticoagulant tube. Red cells were then removed by incubation with red cell lysis buffer. PBMCs were washed and cultured in complete RPMI-1640 medium containing 10% FBS. CD8^+^ T cells were isolated from PBMCs using an EasySep Mouse CD8^+^ T cell Isolation kit and cultured under the same conditions as PBMCs.

### Cell imaging

DC 2.4 cells, as a bone marrow derived and immortalized mouse dendritic cell (DC) line, were obtained from the Department of Biomedical Engineering, Huazhong University of Science and Technology. The cells were cultured in a 12-well plate with 1×10^6^ cells/well overnight. Serum-free 1640 medium was added, followed by 100 µL Thioflavin T (Sigma, 2390-54-7) combined peptidic assembly. After 3 hours of incubation, DCs were washed with phosphate-buffered saline (PBS) and fixed in 4% formaldehyde. After washing, DCs were stained with 10 µM DiI cell membrane dye (D4010; US Everbright, Sayreville, New Jersey, USA). Next, 5 µg/mL Hochest nuclear dye (H4047, US Everbright) was coincubated with DCs, which were then observed under confocal microscope (Leica, Wetzlar, Germany).

### Whole TCL vaccine

From whole TCLs, tumor cells were harvested after washing three times with PBS. Next, pure cells were frozen and thawed five times, and the cell debris was freeze-dried and stored at −20°C to prepare the tumor vaccine.

### Neoantigen peptide preparation

Total RNA was extracted from MC38 tumor cells, and cDNA was obtained using reverse transcription PCR. The purified cDNA was sequenced using Sanger sequencing (Sangon Biotech, Shanghai, China). Next, the Adpgk gene neoantigen peptide was synthesized with 28 amino acids GIPVHLELASMTNMELMSSIVHQQVFPT (Nanjing Top-peptide Biotechnology), and some peptides were labeled with green fluorescent protein (GFP), which was used to track the neoantigen in vitro and in vivo.

### Vaccine administration and presentation in vitro

To determine the delivery efficacy of the vaccines with PMA, DC2.4 cells were cultured in a confocal dish. On the following day, GFP-labeled neoantigen plus CpG adjuvant (NeoV) (20 µM) with PMA carrier was added to the culture medium and cocultured for 2, 12, and 72 hours. The cell nuclei were stained with Hochest (0.5 µg/mL), and fluorescence imaging was performed using a LeicaSP8 laser confocal scanning microscope. To illustrate the ability of neoantigen peptide presentation to elicit CD8^+^ T-cell responses, DC2.4 cells or BMDCs were cocultured with PMA, NeoV, or PMA-NeoV for 24 and 48 hours. The cells were tested for DC maturation. CD8^+^ T cells were extracted from PBMCs and cocultured for 24 hours, and interferon-γ (IFN-γ) secreting CD8^+^ T cells were examined using fluorescence-activated cell sorting (FACS).

To analyze in vivo vaccine delivery, 5-week-old C57BL/6J mice (n=3) were subcutaneously administered with NeoV or PMA-NeoV (50 µg GFP-labeled neoantigen peptide and 0.5 µg CpG per mouse). After 2, 12, and 72 hours, the axillary lymph nodes (LNs) were dissected and used for fluorescence imaging. After 3, 10, and 17 days, PBMCs were acquired from different groups to detect different immune cells.

### MC38 and 4T1 mouse tumor model establishment and treatment

For 4T1 tumor mouse model (n=5), 1×10^5^ 4T1 cells were implanted into the mammary fat pad of female Balb/C mice (5-week old). For MC38 tumor mouse model (n=8), 1×10^5^ MC38 cells or less 0.5×10^5^ MC38 cells were subcutaneously injected into the right flank of male C57BL/6J mice (5-week old). At 5 days after tumor cell implantation, the mice were randomly assigned to different therapeutic groups.

The mice were treated as follows: tumor vaccine, aPD-L1 (clone 10F.9G, BioXcell), and IPI-549 (Catalog No. S8330, Selleckchem). Vaccine treatment was performed on days 5, 10, and 15 after tumor cell implantation, and 1×10^6^ TCL or 50 µg neoantigen peptide combined with 0.5 µg CpG were administered to each mouse in the vaccine treatment group via subcutaneous injection. Two days after the first vaccine treatment, 100 µg aPD-L1 was administered to each mouse in the ICB combined therapy and ICB monotherapy groups via intraperitoneal injection. ICB treatment was performed every 3 days for a total of four times. Two days after the first vaccine treatment, 225 µg IPI-549 was administered to the IPI-549 monotherapy and IPI-549 combined therapy groups at 15 mg/kg using oral gavage daily for 2 weeks.

### Fluorescence-activated cell sorting

After different treatments, BMDCs and PBMCs were harvested and blocked with anti-CD16/32 monoclonal antibody for 10 min. The cells were stained with the corresponding fluorochrome-conjugated antibodies at the proper dilutions. Finally, the cells were examined using a BD FACSCanto II flow cytometer. To detect tumor-infiltrating lymphocytes (TILs), the tumors were minced, digested for 60 min at 37°C in RPMI-1640 with 1 mg/mL collagenase type IV and 1 µg/mL DNAase I, and filtered through a 100-μm nylon cell strainer. The cells were then processed for FACS. For LN analysis, single cells from the tissues were isolated by grinding the LNs with a 40-μm nylon filter. For spleen cell analysis, single cells were isolated in a similar manner as LN analysis, but with an additional step of red cell lysis to remove red cells. Single cells obtained from the spleen or LNs were cocultured with TCLs overnight. After the preparation of single-cell suspensions, they were stained with the appropriate fluorescein isothiocyanate-conjugated antibodies. For the cellular surface markers: the single cells were blocked by incubation with anti-CD16/32 monoclonal antibody. The cells were washed with PBS and stained with the corresponding fluorochrome-conjugated antibodies for 30 min at room temperature in dark. For the intracellular markers, cell suspensions were treated with BD Cytofix/Cytoperm Fixation/Permeabilization Solution (Catalog No. 554714) for 20 min at 4°C and then incubated with corresponding antibodies for 30 min. After then, the cells were analyzed using BD FACSAria III flow cytometry. The detail of the antibodies was listed in the online supplemental table S1.

10.1136/jitc-2021-003564.supp1 Supplementary data

### Statistical analysis

Data analyses were performed using GraphPad Prism V.5.0 (La Jolla, California, USA). Student’s t-test was used to compare continuous variables between two independent groups, and one-way analysis of variance was used to compare continuous variables between more than two groups. For statistical analyses, tumor growth was assessed as the final tumor size after completing the treatment. A p value of <0.05 was considered as statistically significant (*p<0.05, **p<0.01, ***p<0.001, and ****p<0.0001).

## Results

### PMA-trapped tumor vaccine formulation

**Figure 1:**
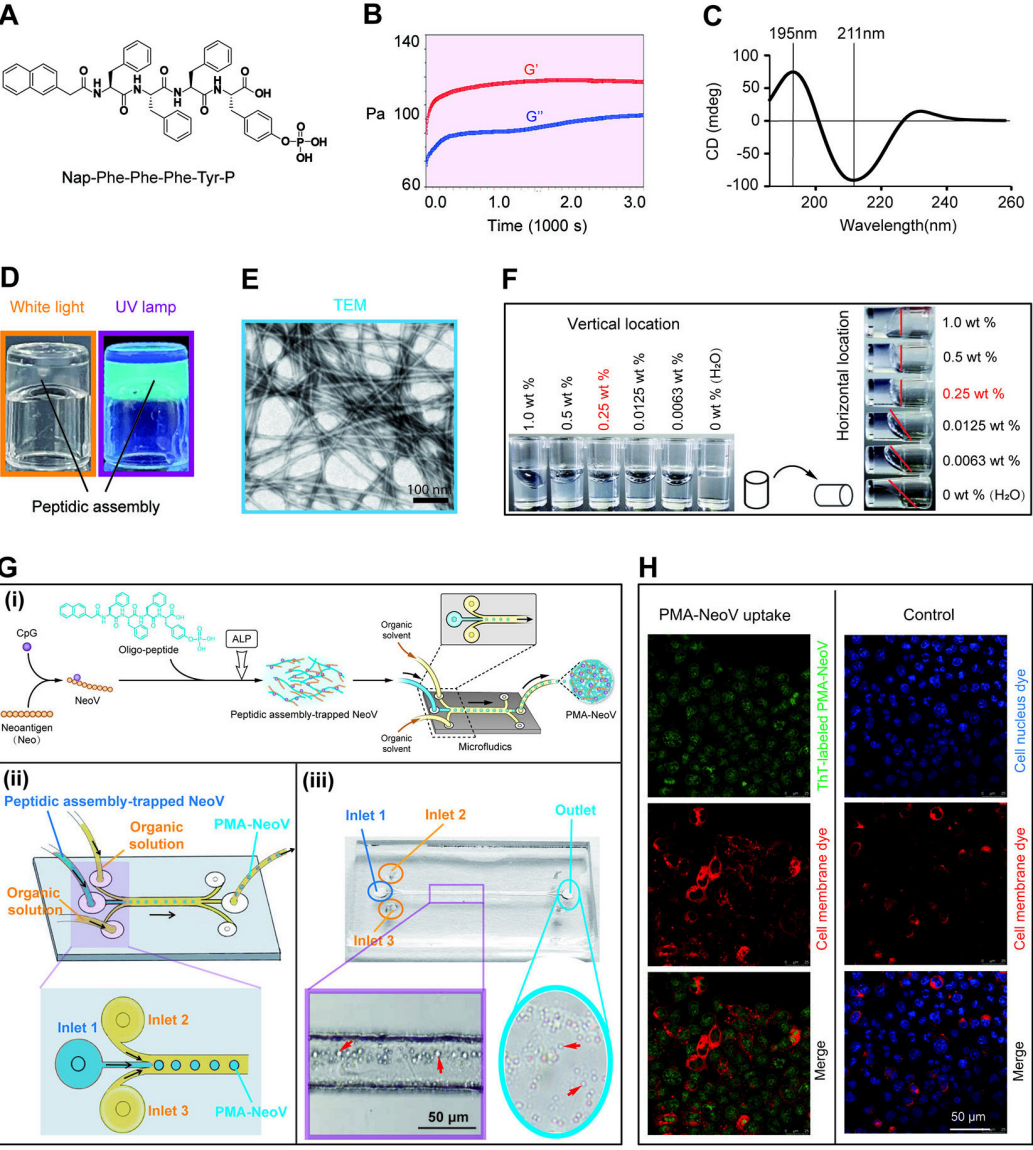
PMA characterization. (A) Chemical structure of the oligopeptide. (B) Mode of dynamic time sweep. (C) Second structure of peptidic assembly. (D) Peptidic assembly morphology. Left: white light image; right: fluorescence image. (E) Transmission electron microscopic (TEM) image of the peptidic assembly. (F) Series concentration of the oligopeptide for the formation of the peptidic assembly. (G) (i) General schematic illustration of the synthesis of PMA-trapped NeoV (PMA-NeoV); (ii) scheme showing the PMA-NeoV-forming process using a microfluidic chip; (iii) images of the microfluidic chip and PMA-NeoV (approximately 3–5 µm in diameter) formation process. (H) Uptake of ThT-labeled PMA-NeoV (green fluorescence) by DCs; the cell membrane is stained with commercial CellVue claret dye (red fluorescence); the cell nucleus is stained with commercial 4′,6-diamidino-2-phenylindole (DAPI) dye (blue fluorescence). PMA, peptidic microarchitecture; PMA-NeoV, PMA-trapped neoantigen vaccine with CpG; ThT, thioflavine-T; UV, ultraviolet.

We designed a new oligopeptide with the following chemical structure: Nap-F-F-F-Y-p (figure 1A). “Nap” at the N terminus and “p” at the C terminus represent the naphthylene and phosphate groups, respectively. The naphthylene group provides π-π stacking to promote peptide assembly, and phosphatase-mediated removal of phosphate groups produce a hydrophobic effect, which can drive rapid peptide assembly.^35^ The mode of dynamic time sweep was further detected. Initially, the value of the storage modulus (G′) was higher than that of the loss modulus (G′′), indicating that the oligopeptides were properly assembled after adding ALP (figure 1B). The secondary structure of peptidic assembly is shown in figure 1C. CD spectrum with a positive peak at 195±10 nm and negative peak at 211±10 nm was defined as a β-sheet structure. The oligopeptide assembly was further characterized. The morphologies of the PMAs were characterized using white light and fluorescence imaging (figure 1D). Thethioflavine-T (ThT) bound to the PMAs produces green fluorescence when illuminated with a ultraviolet lamp. Transmission electron microscopy clearly revealed the formation of peptidic fibrous networks (figure 1E). We further investigated the assembly efficacy of this oligopeptide at different concentrations (0.0063–1 mg/mL). As shown in figure 1F, the critical concentration required to induce fast oligopeptide assembly was 0.25 mg/mL, which was significantly lower than the previously reported assembly concentration, indicating the higher assembly efficacy of our oligopeptide.^24^ Furthermore, a microfluidic chip was used to manufacture the PMA-NeoV (figure 1G). The microarchitectures showed a uniform diameter of 3–5 µm under the optical microscope. The cellular uptake of PMA-NeoV by DCs was further investigated (figure 1H). The PMA-NeoV that was fluorescence-labeled with ThT dye to track their uptake by DC2.4 cells was cocultured with DCs for 4 hours. The fluorescence intensity was significantly higher in DC2.4 cells cocultured with PMA-NeoV than in DCs cultured alone in the control group. Fluorescent PMA-NeoV was mainly in the DC cytoplasm under confocal microscopy, indicating their effective uptake by DCs and potential as vaccine carriers. We also evaluated the release curve of PMA-trapped contents in vitro. The result showed that around 90% of the contents trapped in PMAs were released from day 0 to day 3 (online supplemental figure S1).

### PMA-trapped neoantigen activates immune cells

**Figure 2:**
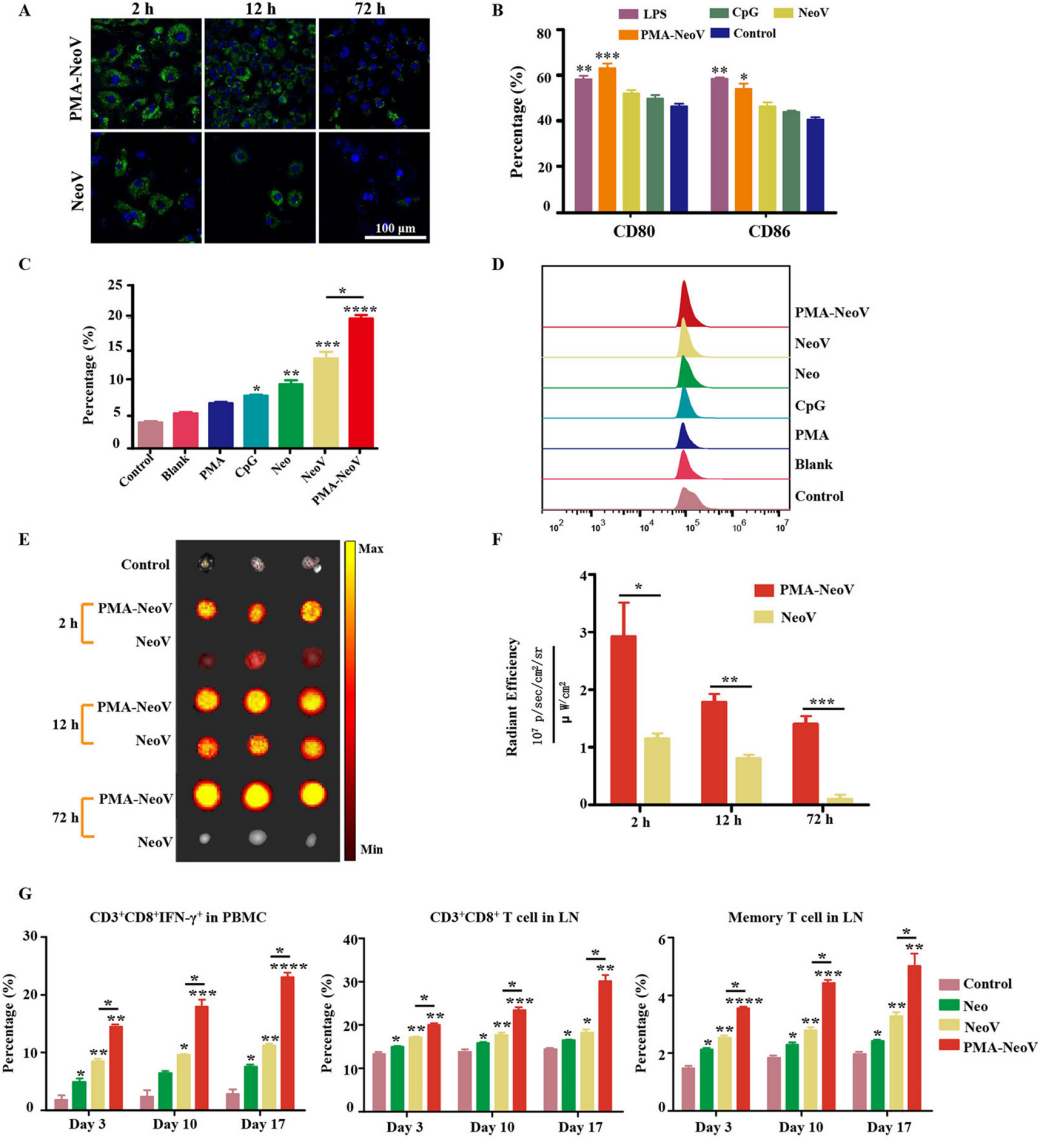
PMA-NeoV system triggered a robust immune response. (A) Confocal imaging of DC2.4 cells after coculture with PMA-NeoV and NeoV for 2–72 hours. The green fluorescence comes from the GFP-labeled Adgpk neoantigen. (B) Percentage of mature DCs of DC2.4 cells after a 24-hour coculture with PMA-NeoV or NeoV. (C and D) Percentage of IFN-γ-secreting CD8^+^ T cells in whole T cells of PBMCs after coculture with activated BMDCs treated with PMA-NeoV or NeoV. (E and F) Fluorescence molecular imaging and fluorescence intensity result of mouse LNs after subcutaneous injection with PMA-NeoV and NeoV. (G) Immune cell changes after PMA-NeoV and NeoV treatments, respectively. BMDC, bone marrow-derived dendritic cell; DC, dendritic cell; GFP, green fluorescent protein; IFN-γ, interferon-γ; IL, interleukin; LNS, lymph nodes; LPS, lipopolysaccharide; Neo, neoantigen only; NeoV, neoantigen vaccine with CpG; PMA-NeoV, PMA-trapped NeoV; PBMCs, peripheral blood mononuclear cells; PMA, peptidic microarchitecture.

To evaluate the uptake efficiency of the PMA-NeoV by antigen-presenting cells (APCs), DC2.4 cells were incubated with either PMA-NeoV or a simple mixture of NeoV. A GFP-labeled neoantigen Adpgk peptide was used, which has been reported as an effective neoantigen epitope in the MC38 tumor model.^13^ Although both forms of neoantigen peptides could be taken up by DC2.4 cells, relatively more PMA-NeoV was taken up (figure 2A). Next, we identified the influence of PMA-NeoV on DC activation. BMDCs and DC2.4 cells were incubated with either PMA-NeoV or NeoV, with LPS as a positive control, for 24 hours, after which the DC maturation was analyzed. The percentage of mature CD80^+^CD86^+^ DCs treated with PMA-NeoV was similar to that in the LPS treatment group and higher compared with that of NeoV and other treatments (figure 2B). The ability of neoantigen presentation to elicit a CD8^+^ T lymphocyte response was also assessed for IFN-γ-secreting CD8^+^ T cells in the whole T cells of PBMCs after coculturing with activated BMDCs or DC2.4 cells treated with PMA-NeoV for 24 hours. The data showed that the control group with only PBMCs, blank group with only BMDCs or DC2.4 cells, and PMA group contained relatively low percentages of IFN-γ-secreting CD8^+^ T cells. The CpG, Neo, NeoV, and PMA-NeoV treatments induced an increase in IFN-γ-secreting CD8^+^ T cells. Compared with NeoV treatment, BMDCs treated with PMA-NeoV resulted in significantly stronger activation of IFN-γ-secreting CD8^+^ T cells at 24 hours (figure 2C, D); similar phenomena were observed on DC2.4 cells (online supplemental figure S2).

Furthermore, we estimated the effect of PMA-NeoV on neoantigen delivery and immune cell activation in vivo. First, the delivery of PMA-NeoV was tracked in vivo using fluorescence molecular imaging. More neoantigen peptides were detected in the draining LNs treated with PMA-NeoV compared with those injected with NeoV from 2 to 12 hours (figure 2E, F). Thereafter, a minimal number of neoantigen peptides were detected in draining LNs treated with NeoV, whereas the neoantigen peptides were still detected after 72 hours when delivered by PMAs. C57BL/6J mice were subcutaneously injected with PMA-NeoV or NeoV on days 1, 6, and 11. To evaluate the in vivo dynamic immune response efficiency of CD8^+^ T cells activated by PMA-NeoV, we analyzed the CD8^+^ T-cell response in PBMCs and LNs at 3 days after each vaccination. A relatively long-lasting and more robust immune response characterized by significantly increased CD8^+^, CD8^+^ IFN-γ^+^, and memory T cells was found in mice injected with PMA-NeoV (figure 2G).

### PMA-trapped TCL vaccine with CpG (TCLV) combined with aPD-L1-inhibited tumor growth

**Figure 3:**
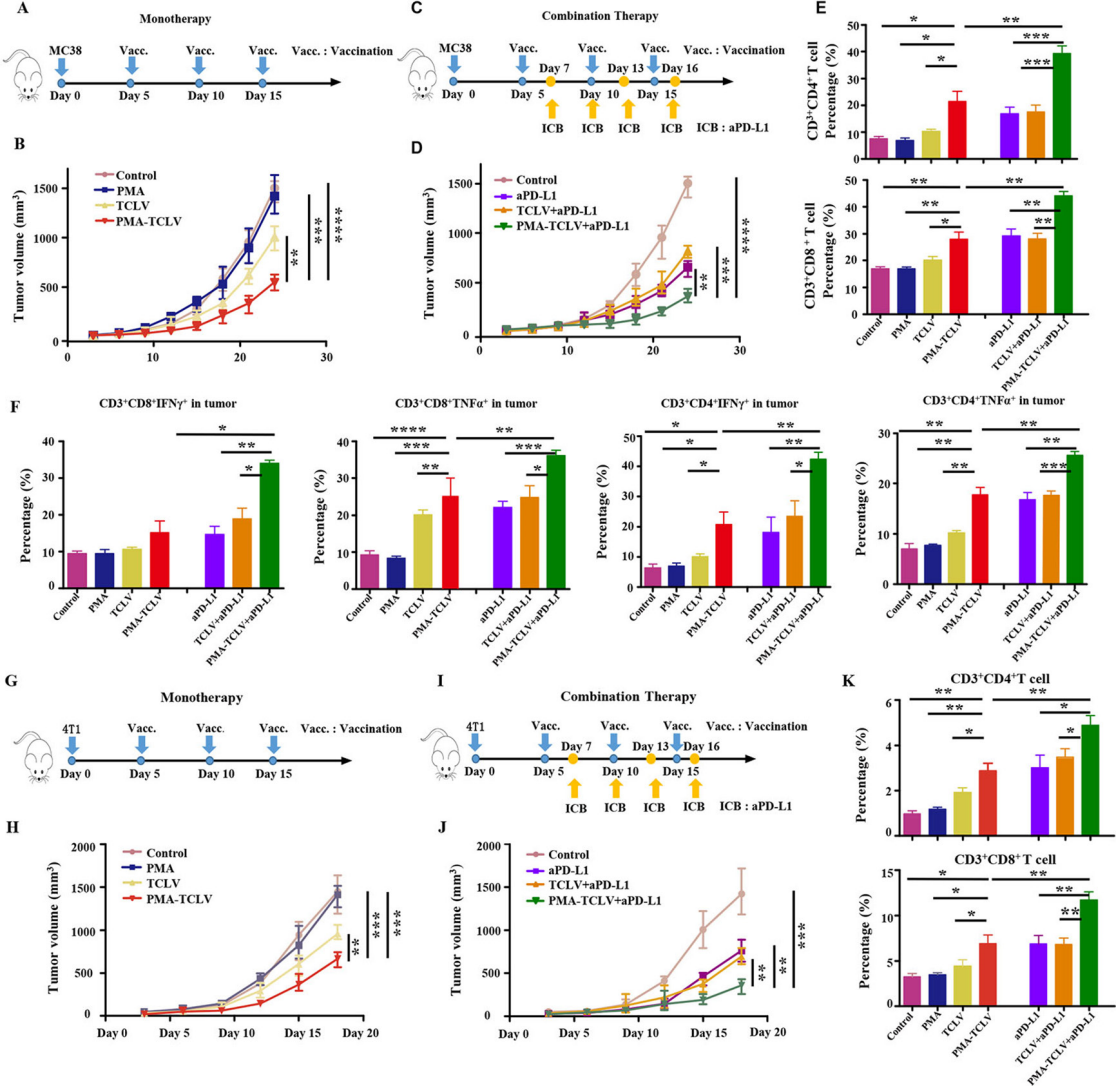
PMA-TCLV slowed tumor growth. (A) Tumor vaccine monotherapy scheme for MC38 tumors. (B) MC38 tumor growth following different monotherapies. (C) Tumor vaccine and aPD-L1 combination therapy scheme for MC38 tumors. (D) MC38 tumor growth after different combination treatments. (E) Lymphocyte percentage in MC38 tumors after different treatments. (F) Percentage of IFN-γ and TNF-α-secreting CD8^+^ and CD4^+^ T cells after different treatments. (G) Tumor vaccine monotherapy scheme for 4T1 tumors. (H) 4T1 tumor growth after different monotherapies. (I) Tumor vaccine and aPD-L1 combination therapy scheme for 4T1 tumors. (J) 4T1 tumor growth after different treatments. (K) Changes in lymphocyte percentages in 4T1 tumors after different treatments. aPD-L1, anti-programmed cell death-ligand 1 antibody; IFN-γ, interferon-γ; PMA, peptidic microarchitecture; PMA-TCLV, PMA-trapped TCLV; TCLV, tumor cell lysate vaccine with CpG; TNF-α, tumor necrosis factor-α.

To evaluate the immunotherapeutic effects of the PMA tumor vaccine, we first tested the TCL as a tumor vaccine in both MC38 and 4T1 tumor mouse models. The therapeutic scheme is shown in figure 3A. Compared with TCLV, PMA-TCLV slowed tumor growth (figure 3B). We found higher levels of programmed cell death-1 (PD-1) expression in immune cells after vaccine treatment in the MC38 tumor model (online supplemental figure S3), suggesting exhaustion of T-cell function. Therefore, combined immunotherapy of a tumor vaccine with aPD-L1 was further performed in the MC38 tumor model (figure 3C). The combination of PMA-TCLV with aPD-L1 efficiently suppressed tumor growth compared with treatment with aPD-L1 alone or in combination with TCLV (figure 3D). To examine the underlying therapeutic mechanism of treatment on the tumor immune microenvironment and immune system, immune cells were analyzed using flow cytometry in the spleen and tumor immune microenvironment. CD3^+^CD4^+^ T and CD3^+^CD8^+^ T cells were significantly increased in tumors treated with PMA-TCLV, showing similar expression levels to those of tumors treated with aPD-L1. However, the combination of PMA-TCLV with aPD-L1 more dramatically augmented the expression level of TILs (figure 3E). Moreover, the key functional cytokines IFN-γ and TNF-α secreting tumor-infiltrating CD3^+^CD4^+^ T and CD3^+^CD8^+^ T cells increased significantly in the group treated with PMA-TCLV and aPD-L1 combination therapy compared with other groups (figure 3F). The percentage of IL-2- and IL-4-secreting CD4^+^ T-helper cells was also significantly augmented (online supplemental figure S4). Furthermore, the number of mature DCs and B cells significantly increased in the LNs, which may have improved the presentation of tumor antigens in mice treated with PMA-TCLV (online supplemental figure S5). The augmented number of mature B cells in mice treated with PMA-TCLV indicated that tumor vaccine also improves humoral immunity. The combined PMA-TCLV and aPD-L1 immunotherapy showed the highest level of positive immune cells in the spleen compared with in the other groups (online supplemental figure S6).

The 4T1 mouse tumor model was also used to confirm the above observation (figure 3G). The data confirmed that TCLV treatment partially inhibited 4T1 tumor growth, which was significantly inhibited by PMA-TCLV treatment during immunization (figure 3H). However, the tumors regrew quickly after vaccination. Hence, to further enhance the therapeutic response, we combined a tumor vaccine with aPD-L1 for the specific experimental protocol shown in figure 3I. Combination immunotherapy with PMA-TCLV and aPD-L1 significantly reduced tumor growth compared with in the groups treated with TCLV and aPD-L1 or either monotherapy (figure 3J). The mechanism underlying the antitumor response of the vaccine and combination therapy was also explored by examining changes of immune cells in the immune system and tumor immune microenvironment. Tumor-infiltrating CD3^+^CD8^+^ and CD3^+^CD4^+^ T cells were greatly increased in mice treated with PMA-TCLV and aPD-L1 compared with in the groups treated with TCLV or control group (figure 3K). The same increase in positive immune cells as that of CD3^+^CD8^+^ and CD3^+^CD4^+^ T cells was also detected in the spleens. Additionally, IFN-γ- and IL-4-secreting CD3^+^CD4^+^ T-helper cells were increased (online supplemental figure S7).

### PMA-NeoV applicability for personalized precision cancer immunotherapy

**Figure 4:**
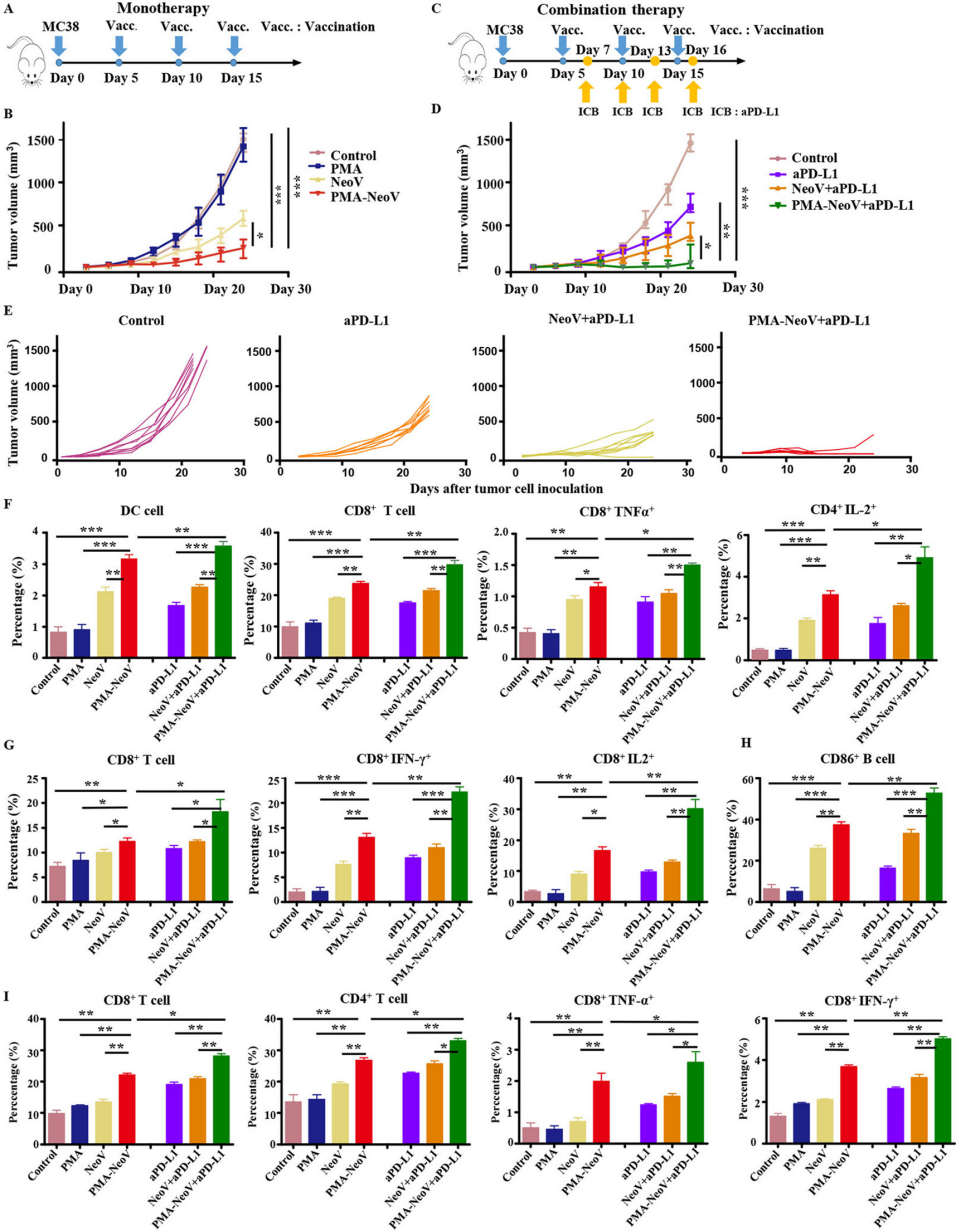
PMA-NeoV and aPD-L1 combination therapy for personalized cancer immunotherapy. (A) Monotherapy scheme for MC38 tumor-bearing mouse model. (B) MC38 tumor growth after monotherapy. (C) PMA-NeoV and aPD-L1 combination therapy scheme for MC38 tumors. (D, E) MC38 tumor growth after different treatments. (F) Immune cell percentage in the lymph nodes of MC38 tumor-bearing mice after different treatments. (G) Immune cell percentage in PBMCs of MC38 tumor-bearing mice after different treatments. (H) Percentage of CD86^+^ mature B cells in PBMCs of MC38 tumor-bearing mice after different treatments. (I) Immune cell percentage in the spleen of MC38 tumor-bearing mice after different treatments. aPD-L1, anti-programmed cell death-ligand 1 antibody; IFN-γ, interferon-γ; IL, interleukin; NeoV, neoantigen vaccine with CpG; PMA, peptidic microarchitecture; PMA-NeoV, PMA-trapped NeoV; TNF-α, tumor necrosis factor-α.

Based on the above findings, we further examined the applicability of PMA-NeoV for personalized immunotherapy. The specific MC38 tumor neoantigen Adpgk was used to formulate the tumor vaccine, and free NeoV and PMA-NeoV were used to treat MC38 tumor-bearing mice (figure 4A). Both PMA-NeoV and free NeoV decreased tumor growth, with more dramatic inhibition of tumor growth by PMA-NeoV (figure 4B). However, although the therapeutic effect was improved, PMA-NeoV did not eradicate the tumor. To further improve the therapeutic effect, aPD-L1 was further combined with vaccine therapy (figure 4C), which dramatically improved the therapeutic effect of combination therapy within 24-day (short “follow-up”) observation (figure 4D). Seven of eight tumors were eradicated in the group treated with a combination of PMA-NeoV and aPD-L1, whereas only one of eight tumors was eradicated in the combination of free NeoV with aPD-L1 (figure 4E). Moreover, the same treatment experiments with the combined PMA-NeoV and aPD-L1 were repeated with less tumor cell inoculation for “long-term” observation for 46 days. The data showed similar therapeutic trends as short “follow-up” observation (online supplemental figure S8).

To explore the effect of the different treatments on the mouse immune system, immune cells in the draining LNs, PBMCs, and spleens were analyzed using flow cytometry. First, a significant increase in mature DCs was detected in the LNs (figure 4F), indicating efficient vaccine delivery to LNs. Furthermore, CD3^+^CD8^+^ T cells were significantly increased in mice treated with PMA-NeoV and aPD-L1. In addition, the TNF-α and IL-2 cytokine-secreting CD3^+^CD8^+^ T effector cells and CD3^+^CD4^+^ T-helper cells greatly increased (figure 4F). Second, similar results were observed for increased CD3^+^CD8^+^ T cells in PBMCs treated with PMA-NeoV and aPD-L1 (figure 4G). Moreover, the percentage of IFN-γ- and IL-2-secreting CD3^+^CD8^+^ T cells significantly increased in mice treated with combined PMA-NeoV and aPD-L1 therapy. Third, we detected an increase in the number of mature CD86^+^ B cells in the PBMCs of mice treated with PMA-NeoV, indicating the augmentation of antigen presentation ability and humoral immunity in PBMCs (figure 4H). Finally, in the spleen, PMA-NeoV treatment significantly promoted the maturation of lymphocytes, including CD3^+^CD8^+^ T cells and CD3^+^CD4^+^ T-helper cells. Particularly, the number of T effector cells secreting functionally cytotoxic cytokines, such as TNF-α and IFN-γ, greatly increased after treatment with PMA-NeoV plus aPD-L1 (figure 4I).

### Macrophage polarization illustrated functional mechanism underlying tumor vaccine therapy

**Figure 5:**
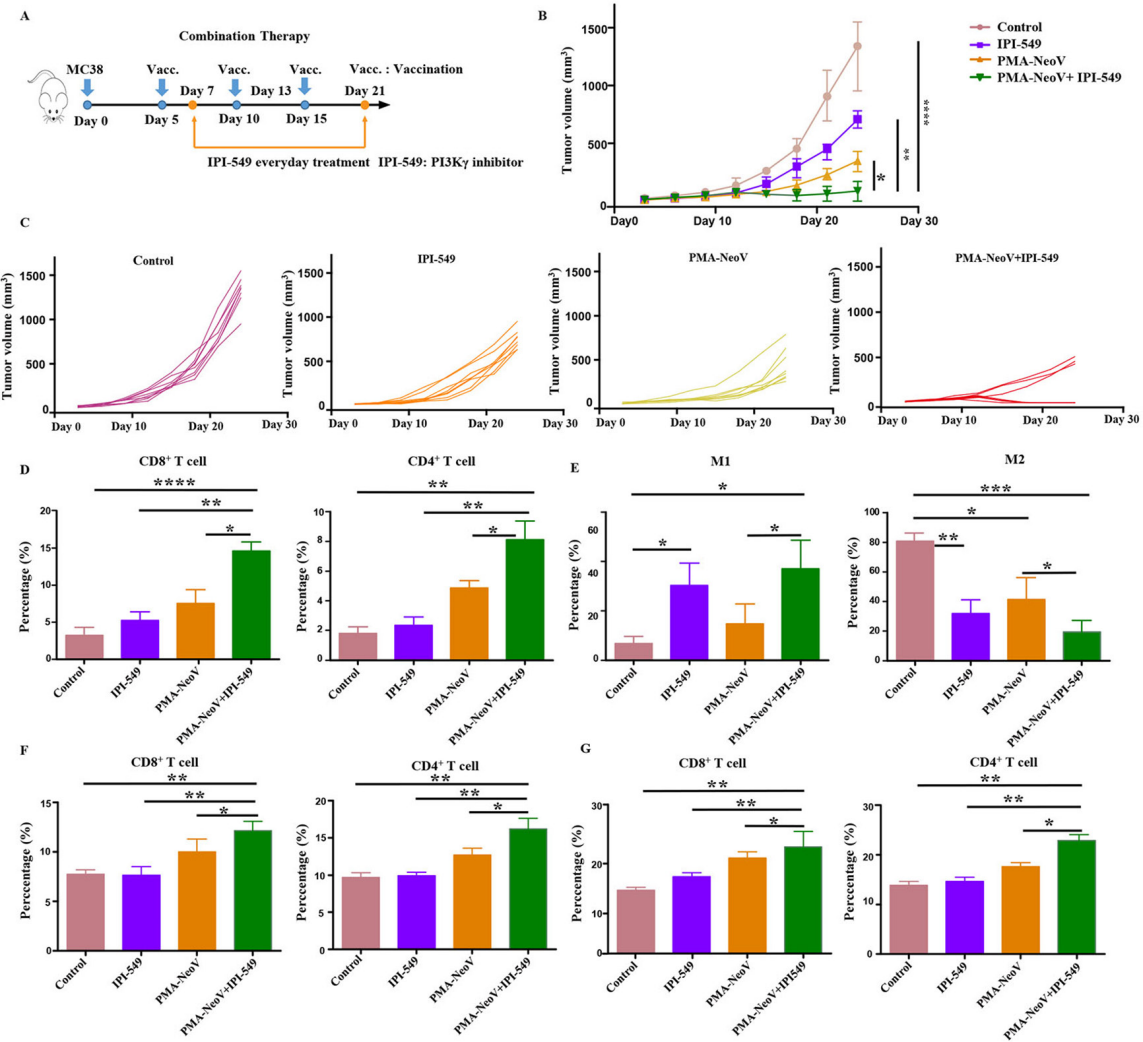
PMA-NeoV and PI3Kγ inhibitor combination therapy. (A) The scheme of tumor vaccine and PI3Kγ inhibitor combination therapy. (B, C) MC38 tumor growth after tumor vaccine and PI3Kγ combination therapy. (D) Lymphocyte percentage in MC38 tumors after different treatments. (E) Percentage of the different states of macrophages in MC38 tumors after different treatments. (F) Lymphocyte percentage in the spleen of mice inoculated with MC38 tumors with different treatments. (G) Lymphocyte percentage in the lymph nodes of mice inoculated with MC38 tumors after different treatments. IPI-549, PI3Kγ inhibitor; PMA, peptidic microarchitecture; PI3Kγ, phosphatidylinositol 3-kinase γ; PMA-NeoV, PMA-trapped NeoV; NeoV, neoantigen vaccine with CpG.

We found that M2 macrophages showed little change during treatment with PMA-NeoV (online supplemental figure S9). Therefore, we hypothesized that switching of M2 macrophages to M1 macrophages would enhance the curative effect of PMA-NeoV. IPI-549 and PMA-NeoV were applied to the MC38 tumor mouse model to examine this hypothesis (figure 5A). The data showed that IPI-549 alone slowed tumor growth to some degree. However, the combination of IPI-549 with PMA-NeoV dramatically suppressed tumor progression and eradicated five of eight tumors within 24-day (short “follow-up”) observation (figure 5B, C), indicating that IPI-549 synergized with PMA-NeoV to effectively kill tumor cells. Moreover, the treatment experiments with combined PMA-NeoV and IPI-549 were repeated with less tumor cell inoculation for “long-term” observation for 46 days. The data showed similar therapeutic trends as short “follow-up” observation (online supplemental figure S10). We further analyzed immune cells in the tumors at 2 days after the last vaccination. First, we detected a significant increase in infiltrating CD3^+^CD8^+^ and CD3^+^CD4^+^ T cells in the combination IPI-549 with PMA-NeoV group compared with that in the other groups (figure 5D). M1 macrophages were increased and M2 macrophages were found decreased in the IPI-549 and the combined IPI-549 and PMA-NeoV treatment groups (figure 5E).

The influence of IPI-549 and PMA-NeoV combination therapy on the immune system was further investigated. The immune cell components of the LNs and spleens were analyzed. PMA-NeoV and IPI-549 synergistically improved systemic immunity by increasing the number of functional lymphocytes, including CD3^+^CD8^+^ T cells and CD3^+^CD4^+^ T cells in both the LNs (figure 5F) and spleens (figure 5G). Moreover, the expression of myeloid-derived suppressor cells in the tumor, spleen, and LNs was also decreased after treatment with IPI-549, PMA-NeoV, and IPI-549+PMA NeoV (online supplemental figure S11). These data suggested that the combination of PMA-NeoV and PI3Kγ inhibitor enhanced both innate and adaptive immunogenicity.

## Discussion

**Figure 6:**
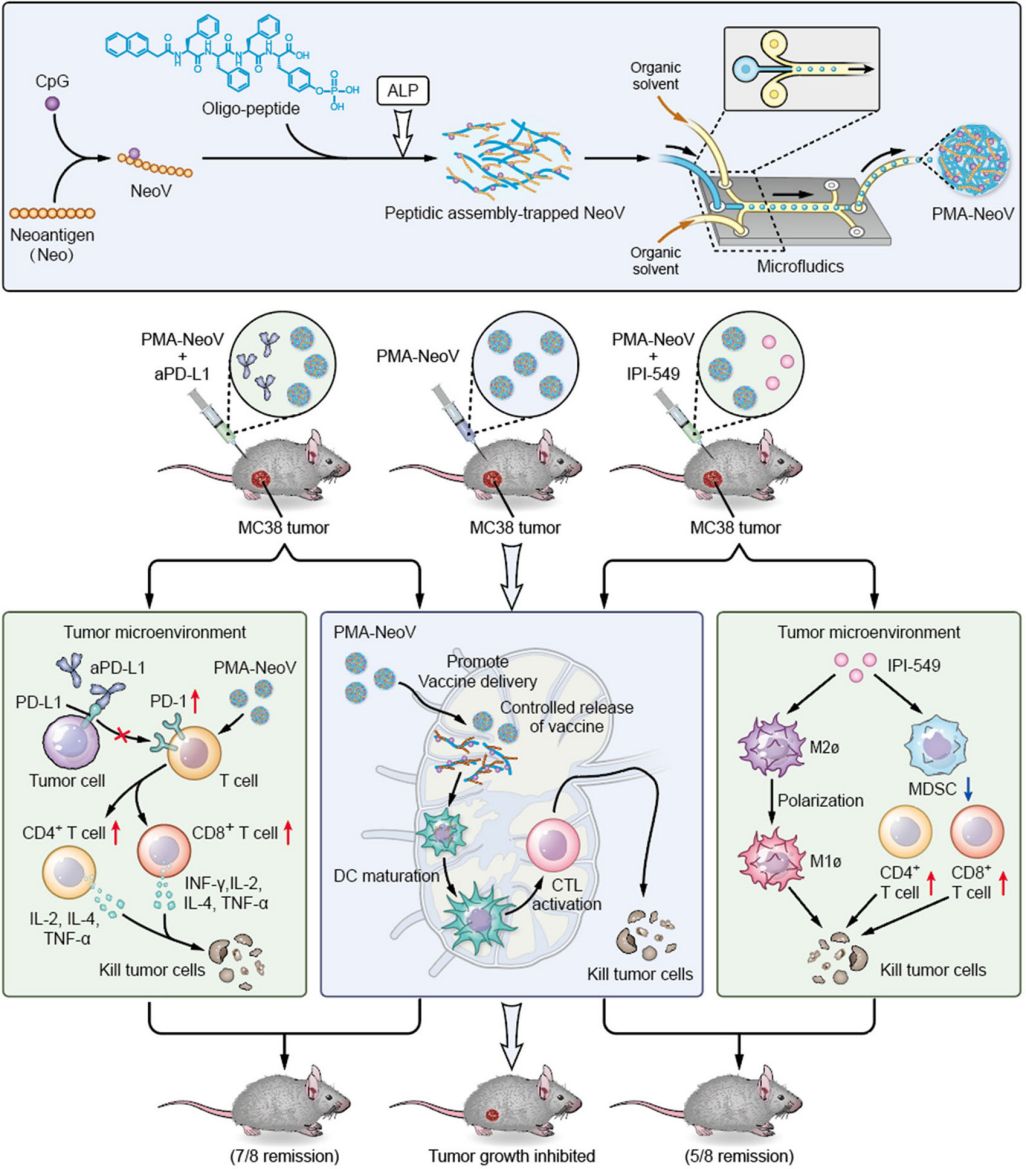
Schematic illustration of synthesis of the PMA-trapped tumor vaccine with enhanced immunogenicity that can eradicate tumors in combination with immune checkpoint inhibitor or PI3Kγ inhibitor. aPD-L1, anti-programmed cell death-ligand 1 antibody; ALP, alkaline phosphatase; DC, dendritic cell; CTK, cytotoxic T lymphocyte; IFN-γ, interferon-γ; IL, interleukin; MDSCs, myeloid-derived suppressor cells; NeoV, neoantigen vaccine with CpG; PD-1, programmed cell death-1; PI3Kγ, phosphatidylinositol 3-kinase γ; PMA, peptidic microarchitecture; TNF-α, tumor necrosis factor-α.

We developed a PMA tumor vaccine delivery carrier with improved assembly efficacy and biosafety. A highly effective assembly can dramatically improve the robustness of oligopeptides as vaccine carriers, enabling multiple target-carrying. Moreover, microfluidics was introduced to precisely produce uniform PMA particles. The PMA formulation efficiently delivered tumor vaccines and sustained neoantigen presentation to APCs to achieve a long-term immune response, triggering systemic immunity against tumor neoantigens and promoting infiltration of functional immune cells, such as CD8^+^ T, CD4^+^ T, and B cells, to inhibit both MC38 and 4T1 tumor growth. Therefore, our PMA-NeoV system shows potential for developing advanced tumor vaccine-based therapies. Importantly, we also provide alternative promising treatment strategies using combinations of PMA-NeoV with aPD-L1 or PI3Kγ inhibitor, showing synergistic immunotherapeutic effects and effectively eradicating most tumors in mice with no observable toxicity, including no obvious body weight loss or major organ damage (figure 6 and online supplemental figures S12–S15).

Previous studies reported the excellent biosafety and biocompatibility of peptide-assembled materials in vitro and in vivo,^36^ which greatly promote the bioapplication of peptidic materials. Peptidic assemblies have been widely examined because of their easy preparation and superior drug-loading capability.^37^ We previously demonstrated that the assembly efficacy of oligopeptides is primarily determined by phenylalanine-mediated aromatic–aromatic interactions. Thus, to effectively formulate peptidic assemblies, we optimized the design of the chemical structure of oligopeptides. We used three phenylalanines as the functional unit of assembly and obtained an effective peptidic assembly, compared with the two-phenylalanine assembly unit, which has been reported previously.^35 36^ In most studies, peptidic assemblies were directly injected into the body, which will inevitably form various gels with different sizes and shapes in vivo, resulting in poor reproducibility of delivery and release of contents packaged into the peptidic assembly. To overcome this limitation, a microfluidic chip was used to produce PMAs with a uniform size and shape, achieving more precise control of the delivery and release of vaccines, adjuvants, and drugs packaged into the PMAs.

Our improved formulation also required high-quality neoantigens to induce a robust immune response. In early tumor vaccine studies and in our study, whole TCLs were used as a neoantigen source to promote immunogenicity against cancer cells. However, the content of tumor neoantigens in the cell lysates is complex, resulting in a very limited immune response. Recent advances in genomic sequencing and mass spectrometry technologies enable the detection of high-quality tumor-specific neoantigens. We used the specific MC38 tumor neoantigen Adpgk to formulate the tumor vaccine, PMA-NeoV, which can more efficiently activate systemic immunity and suppress tumor progression. The strategy as recent blood-free tumor DNA detection technique enriched tumor cell epitopes and amplified the specific systemic immune response, enabling more precise and personalized tumor therapy and facilitating the development of neoantigen-based tumor vaccines.

Most therapeutic tumor vaccines fail to eradicate tumors, and similar phenomena were observed in this study. Tumor immune escape results from the limited number of immune cells that fight against tumor cells and the number of immune cells exhausted or suppressed by cancer cells.^38^ Therefore, combination therapeutic strategies are required to further enhance the antitumor activity of tumor vaccines. The first strategy uses ICB, which has been combined with many tumor vaccines to treat cancer in preclinical and clinical trials. Additionally, the combination of PMA-NeoV with aPD-L1 had a strong therapeutic effect, eradicating seven of eight inoculated MC38 tumors in mice. Moreover, we used a combination of a tumor vaccine with a PI3Kγ inhibitor to treat MC38 tumors, which not only dramatically inhibited tumor growth but also eradicated five of eight inoculated tumors. PI3Kγ inhibitors suppress tumor growth mainly by switching macrophages from the immune suppressed state to the active state and by priming a more robust lymphocyte response.^32 33^ Selective inhibition of PI3Kγ, which is highly expressed in myeloid cells, can restore ICB sensitivity.^32^ The PI3Kγ inhibitor synergizes with PD-L1 blockade to improve the antitumor response, and hence increase the survival of tumor-bearing mice in a CD8^+^ T lymphocyte-dependent manner.^39^ Therefore, the combination of PD-L1 blockade and PI3Kγ inhibitor with the PMA-based tumor neoantigen vaccine represents a promising and effective treatment method. Immune-related adverse side effects should be considered for combined immunotherapy.

In conclusion, we present an effective approach for improving tumor vaccine immunogenicity by developing a novel PMA vaccine carrier. When combined with ICB or PI3Kγ inhibitor, PMA-NeoV effectively eradicated MC38 tumors in vivo. Our study provides an important foundation for the clinical improvement of cancer vaccine applications and combined therapies.

## Data Availability

All data relevant to the study are included in the article or uploaded as supplemental information.
