# Supplementary material for: Peptidic microarchitecture-trapped tumor vaccine combined with immune checkpoint inhibitor or PI3Kγ inhibitor can enhance immunogenicity and eradicate tumors
Source: J Immunother Cancer. 2022 Feb 25;10(2):e003564. doi: 10.1136/jitc-2021-003564 (PMC8883272; doi:10.1136/jitc-2021-003564)
Supplement: Supplementary data [file jitc-2021-003564supp001.pdf]

Supplementary Information

Peptidic micro-architecture-trapped tumor vaccine combined with immune checkpoint inhibitor or PI3Kγ inhibitor can enhance immunogenicity and eradicate tumors

Yang Du[#,1,3], Ye Liu[#,2], Di Wang[#,4], Hua Bai[4], Zhijie Wang[4], Xiran He[1,4], Pei Zhang[1,4], Jie Tian[*,1,3,5,6], Jie Wang[*,4]

[1] CAS Key Laboratory of Molecular Imaging, Beijing Key Laboratory of Molecular Imaging, the State Key Laboratory of Management and Control for Complex Systems, Institute of Automation, Chinese Academy of Sciences, Beijing, 100190, China.

[2] Institute of Medical Biology, Chinese Academy of Medical Sciences and Peking Union Medical College, Kunming, Yunnan, 650000, China.

[3] The University of Chinese Academy of Sciences, Beijing 100049, China

[4]Department of Medical Oncology, National Cancer Center/National Clinical Research Center for Cancer/Cancer Hospital, Chinese Academy of Medical Sciences & Peking Union Medical College, Beijing, China

[5]Beijing Advanced Innovation Center for Big Data-Based Precision Medicine, School of Medicine, Beihang University, Beijing, 100191, China

[6]School of Life Science and Technology, Xidian University, Xi'an, Shaanxi, 710071, China

# Yang Du, Ye Liu and Di Wang contribute equally to this work

* Correspondence to Dr. Jie Wang, zlhuxi@163.com; Dr. Jie Tian, jie.tian@ia.ac.cn

Supplementary experimental methods

Evaluation of PMA release

To evaluate the release curve of PMA, 1 mg/mL assembly peptides were dissolved with phosphate buffer solution. ALP-labeled antibodies (Proteintech, Rosemont, IL, USA) were selected as the loading content to evaluate the release curve of PMA. ALP-labeled antibodies (5 μL) were added to 1 mL assembly peptide solution to formulate PMA. PMA was incubated with 1 mL phosphate buffer solution at 37°C with shaking. The supernatants were harvested at series days. ALP-labeled antibodies in the supernatant were quantified using an alkaline phosphatase kit (Beyotime, Shanghai, China).

Du Y, *et al. J Immunother Cancer* 2022; 10:e003564. doi: 10.1136/jitc-2021-003564

Supplementary table

Supplementary Table 1

Antibody Name	Conjugate	Clone Nunber	Identifier	Source
CD45	Efluor 450	A20	50-162-98	Thermo Fisher
CD3	PerCP-CY5.5	17A2(RUO)	560527	BD
CD4	APC-eFluor @780	RM4-5	47-0042-82	Thermo Fisher
CD8	PE-CY7	53-6.7(RUO)	561097	BD
PD-1	PE	RMP1-30 (RUO)	566831	BD
CD11b	APC	M1/70 (RUO)	553312	BD
F4/80	PE-CY7	BM8	25-4801-82	Thermo Fisher
Gr-1	APC-CY7	RB6-8C5 (RUO)	557661	BD
CD11C	BV510	HL3 (RUO)	562949	BD
IFN-γ	APC	XMG1.2	562018	BD
IL-2	eFluor 450	JES6-5H4	48-7021-82	Thermo Fisher
IL-4	PE	11B11	562044	BD
TNF-α	PE-Cyanine7	MP6-XT22	561041	BD
CD86 (B7-2)	PE	GL1(RUO)	560582	BD

Du Y, *et al. J Immunother Cancer* 2022; 10:e003564. doi: 10.1136/jitc-2021-003564

Supplementary Figures S1-15 and figure legends

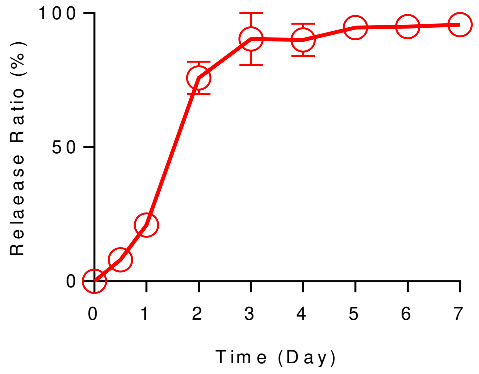

Supplementary Figure S1. Release curve of PMA.

PMA, peptidic micro-architecture.

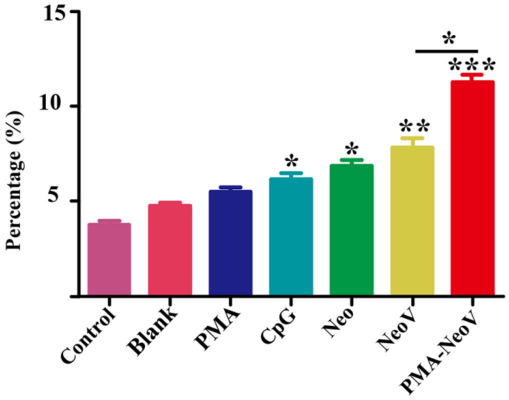

Supplementary Figure S2. The percentage of IFN-γ-secreting CD8$^+$ T cells in the whole CD8$^+$ T cells of PBMCs after co-culture with activated DC2.4 cells with different treatments for 48 h.

PMA, peptidic micro-architecture; Neo, neoantigen; NeoV, neoantigen vaccine with CpG; PBMCs, peripheral blood mononuclear cells; DCs, dendritic cells.

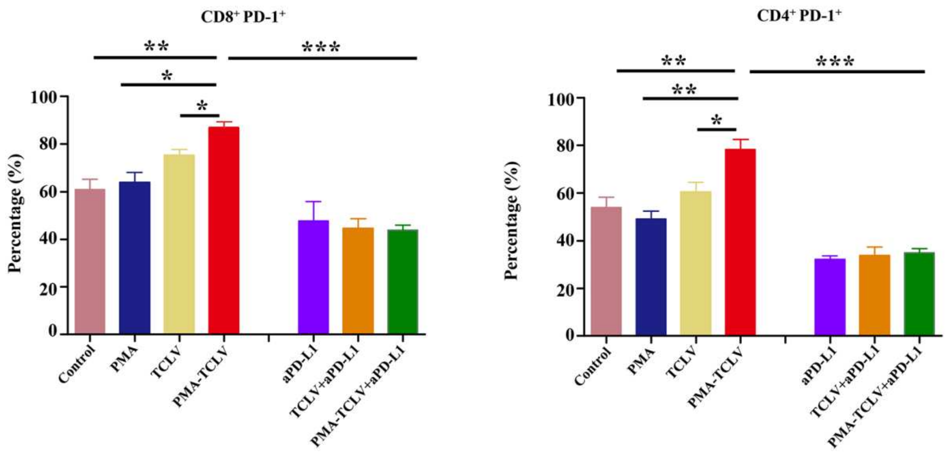

Supplementary Figure S3. The percentage of PD-1 expressing tumor-infiltrating lymphocytes in MC38 tumors after different treatments.

PMA, peptidic micro-architecture; TCLV, tumor cell lysate vaccine with CpG; PD-1, programmed cell death-1; aPD-L1, anti-programmed cell death-ligand 1 antibody.

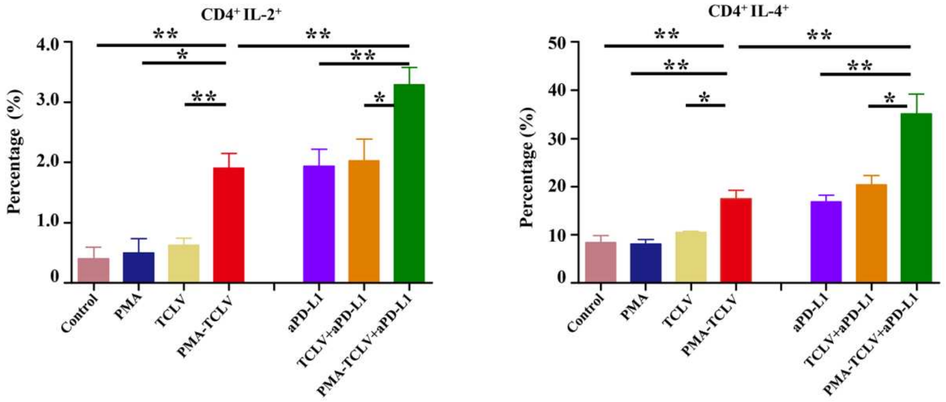

Supplementary Figure S4. The percentage of IL-2 secreting CD3$^+$CD4$^+$ T helper cells in MC38 tumors after different treatments.

PMA, peptidic micro-architecture; TCLV, tumor cell lysate vaccine with CpG; aPD-L1, anti-programmed cell death-ligand 1 antibody; IL, interleukin.

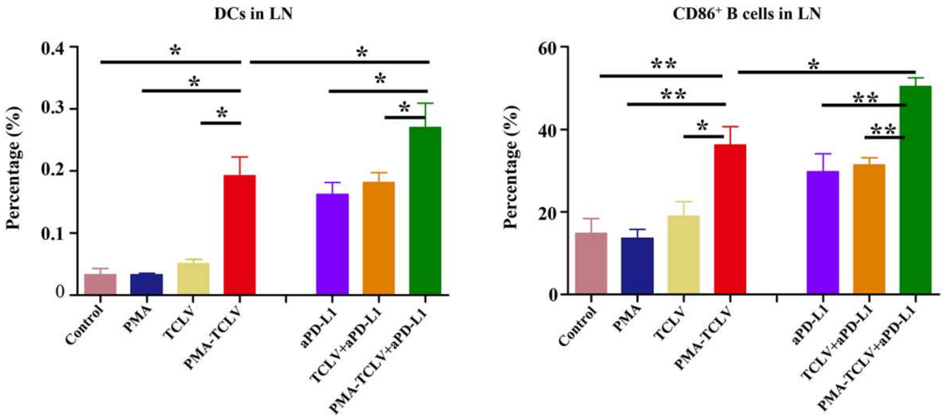

Supplementary Figure S5. The percentage of DCs and mature B cells in LNs of MC38 tumor-bearing mice after different treatments.

DCs, dendritic cells; LN, lymph node; PMA, peptidic micro-architecture; TCLV, tumor cell lysate vaccine with CpG; aPD-L1, anti-programmed cell death-ligand 1 antibody.

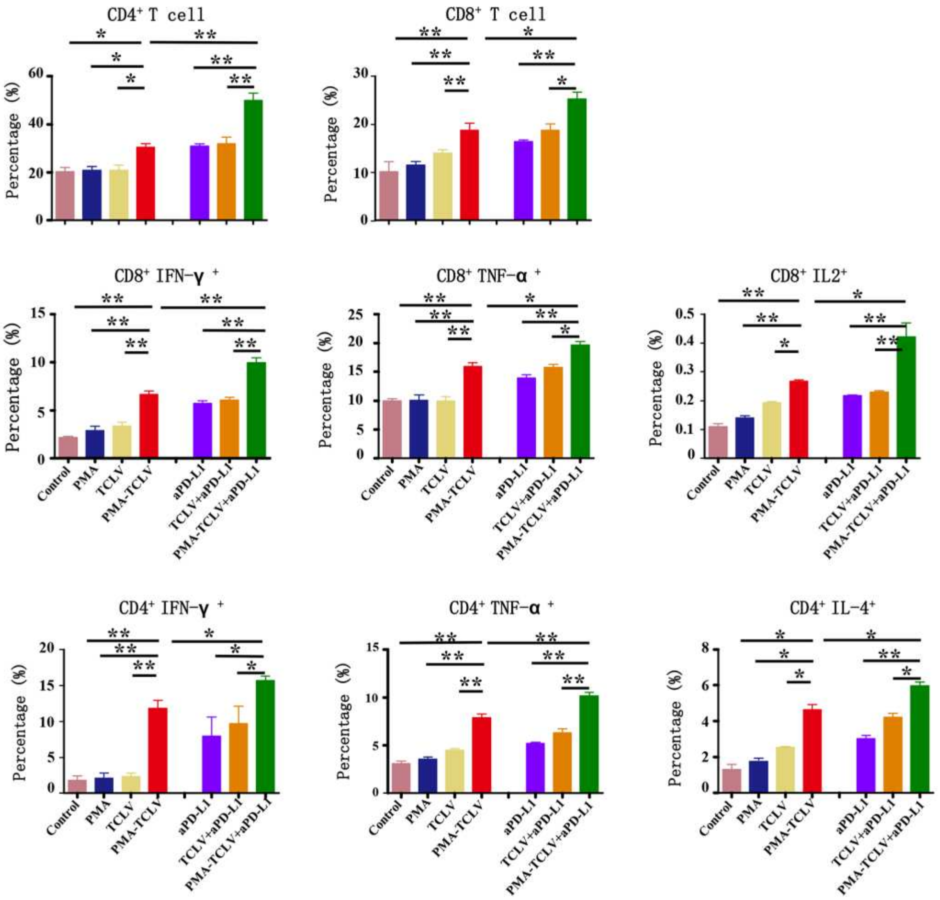

Supplementary Figure S6. The percentage of immune cells in the spleen of MC38 tumor-bearing mice after different treatments.

PMA, peptidic micro-architecture; TCLV, tumor cell lysate vaccine with CpG; IL-4, interleukin-4; IFN-γ, interferon-γ; aPD-L1, anti-programmed cell death-ligand 1 antibody, TNF-α, tumor necrosis factor -α.

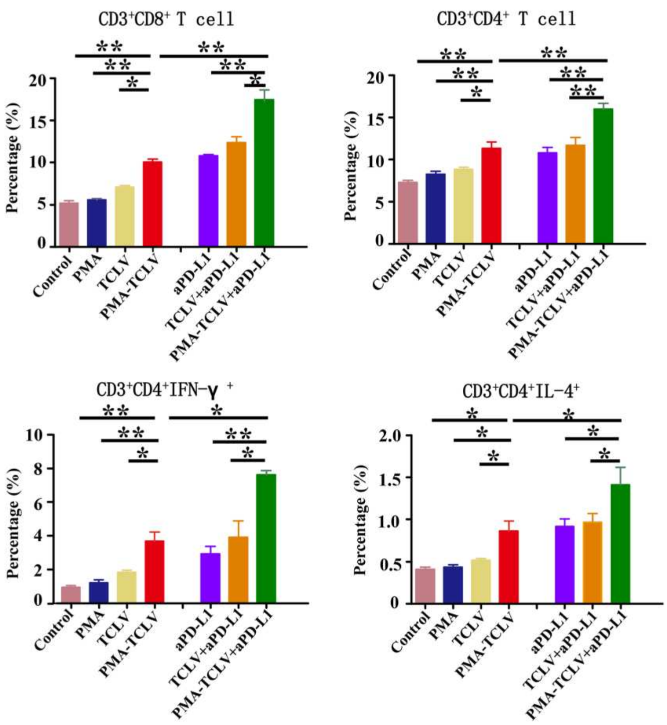

Supplementary Figure S7. The percentage of CD3$^+$CD8$^+$ T and CD3$^+$CD4$^+$ T cells, and IFN-γ and IL-4-secreting cell percentage of CD3$^+$CD4$^+$ T helper cells in the spleens of 4T1-tumor bearing mice after different treatments.

PMA, peptidic micro-architecture; TCLV, tumor cell lysate vaccine with CpG; IL-4, interleukin-4; IFN-γ, interferon-γ; aPD-L1, anti-programmed cell death-ligand 1 antibody.

Du Y, *et al. J Immunother Cancer* 2022; 10:e003564. doi: 10.1136/jitc-2021-003564

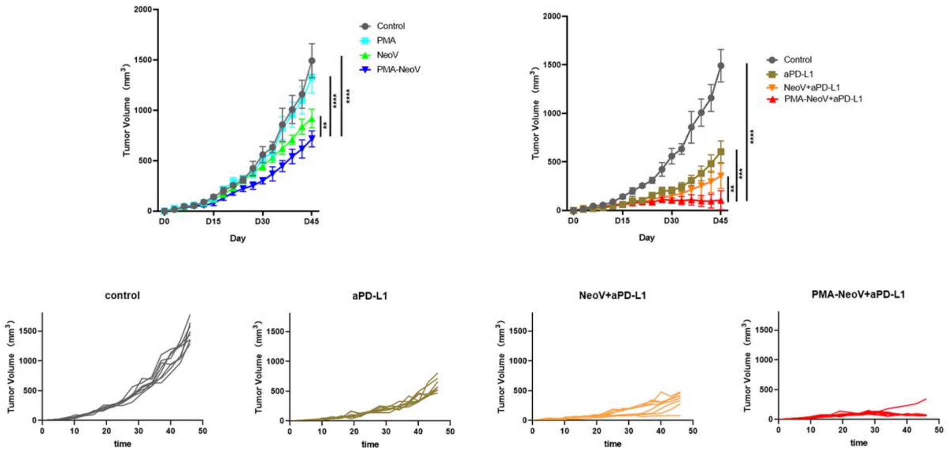

Supplementary Figure S8. The 46-day long "follow-up" observation of therapeutic efficacy with PMA-NeoV and aPD-L1 combination therapy.

PMA-NeoV, peptidic micro-architecture (PMA)-trapped NeoV

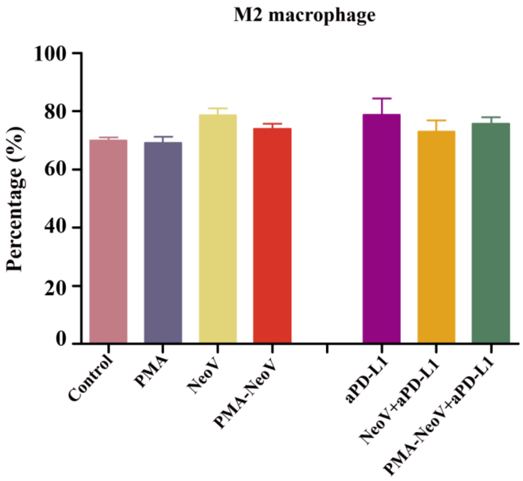

Supplementary Figure S9. The percentage of M2 macrophages in MC38 tumors after different treatments.

PMA, peptidic micro-architecture; NeoV, neoantigen vaccine with CpG; aPD-L1, anti-programmed cell death-ligand 1 antibody.

Du Y, *et al. J Immunother Cancer* 2022; 10:e003564. doi: 10.1136/jitc-2021-003564

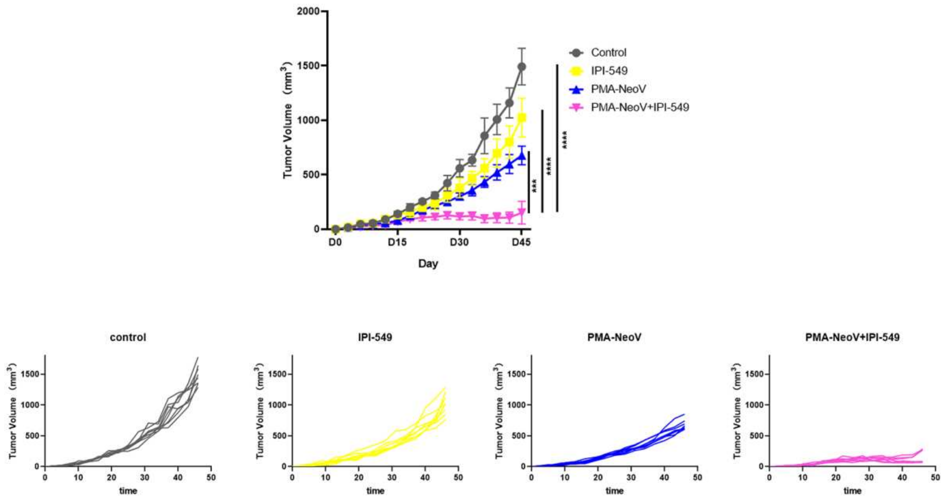

Supplementary Figure S10. The 46-day long “follow-up” observation with PMA-NeoV and IPI-549 combination therapy.

PMA-NeoV, peptidic micro-architecture (PMA) trapped NeoV.

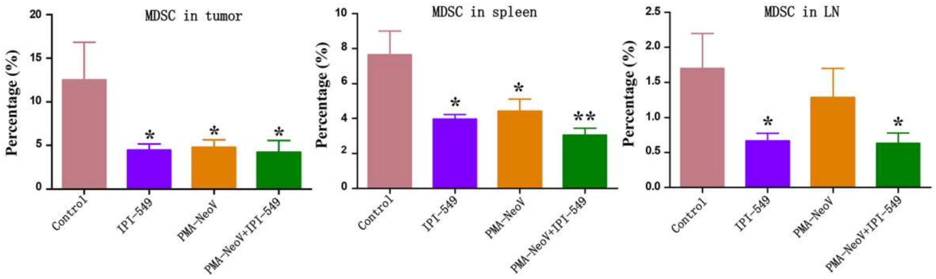

Supplementary Figure S11. The percentages of MDSCs in MC38 tumor bearing mice after different treatments.

MDSC, myeloid-derived suppressor cell; NeoV, neoantigen vaccine with CpG; PMA-NeoV, peptidic micro-architecture trapped NeoV.

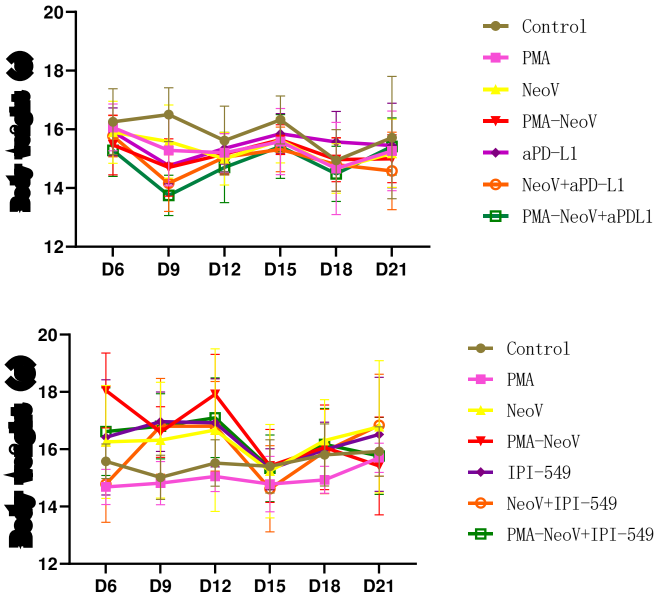

Supplementary Figure S12. Body weight changes with different treatments during 24-day observation.

PMA, peptidic micro-architecture; NeoV, neoantigen vaccine with CpG; PMA-NeoV, PMA trapped NeoV; aPD-L1, anti-programmed cell death-ligand 1 antibody.

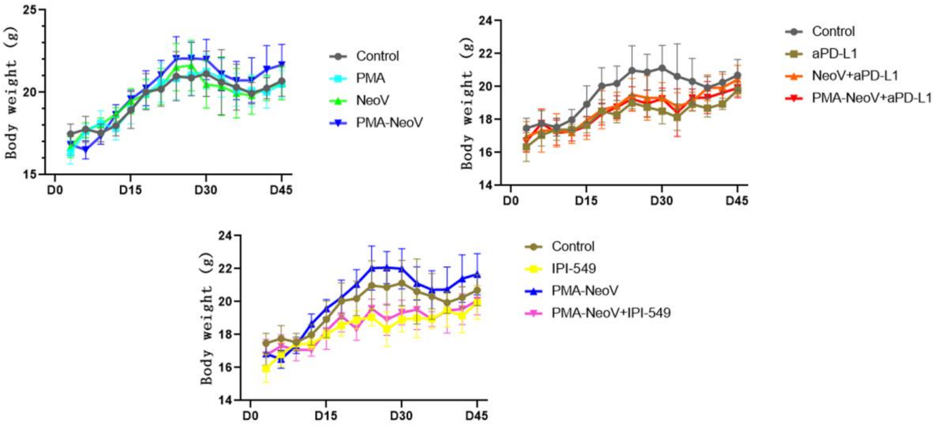

Supplementary Figure S13. Body weight changes with different treatments during 46-day “follow-up” observation.

PMA, peptidic micro-architecture; NeoV, neoantigen vaccine with CpG; PMA-NeoV, PMA trapped NeoV; aPD-L1, anti-programmed cell death-ligand 1 antibody.

Du Y, *et al. J Immunother Cancer* 2022; 10:e003564. doi: 10.1136/jitc-2021-003564

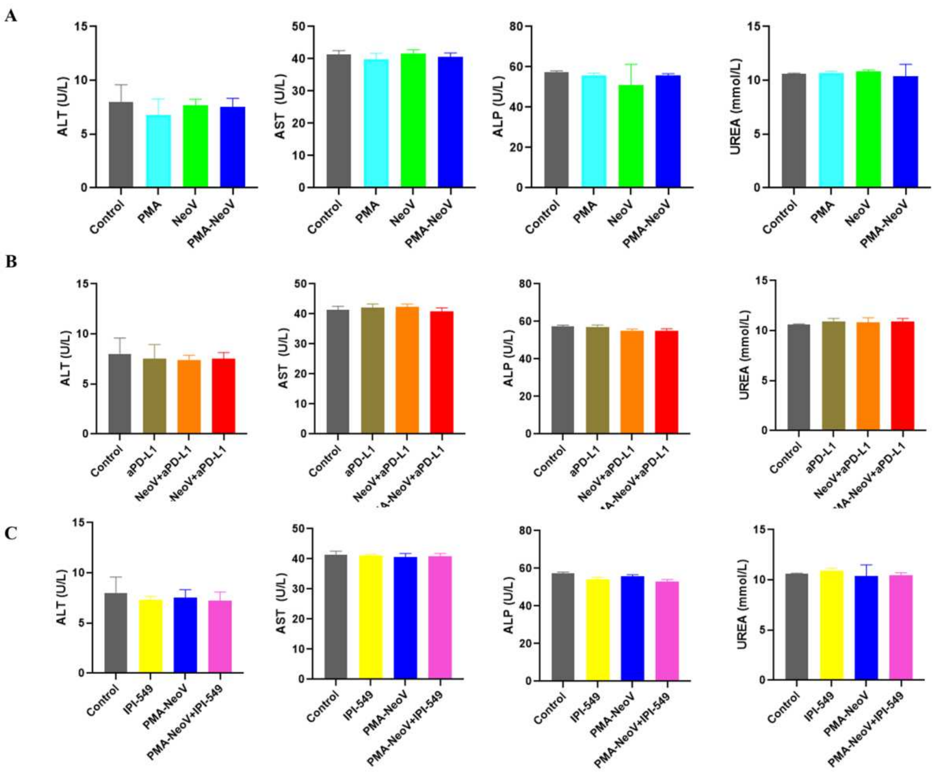

Supplementary Figure S14. Liver and kidney function evaluation after different treatments.

ALT, Alanine transaminase; AST, Aspartate transaminase; ALP, alkaline phosphatase; PMA, peptidic micro-architecture; NeoV, neoantigen vaccine with CpG; PMA-NeoV, PMA trapped NeoV; aPD-L1, anti-programmed cell death-ligand 1 antibody.

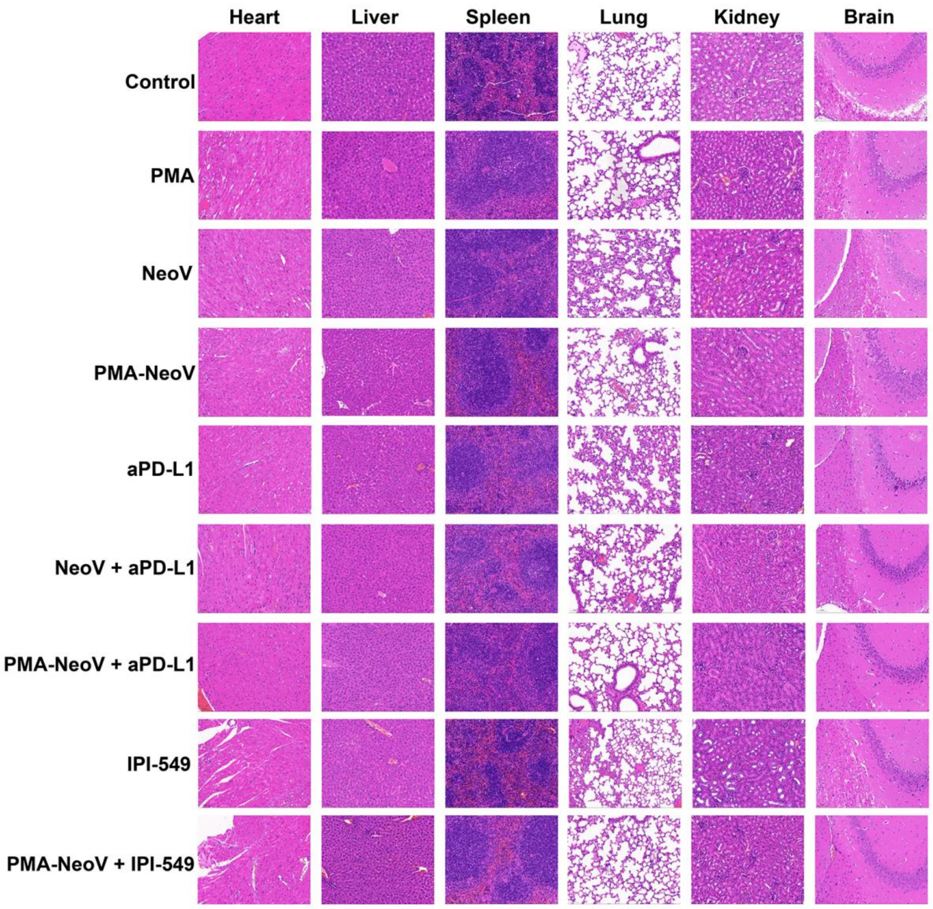

Supplementary Figure S15. Histological analysis of major organs after different treatments.

PMA, peptidic micro-architecture; NeoV, neoantigen vaccine with CpG; PMA-NeoV, PMA trapped NeoV; aPD-L1, anti-programmed cell death-ligand 1 antibody.

Du Y, *et al. J Immunother Cancer* 2022; 10:e003564. doi: 10.1136/jitc-2021-003564
